# Antioxidative and Anti-Inflammatory Properties of Cannabidiol

**DOI:** 10.3390/antiox9010021

**Published:** 2019-12-25

**Authors:** Sinemyiz Atalay, Iwona Jarocka-Karpowicz, Elzbieta Skrzydlewska

**Affiliations:** Department of Analytical Chemistry, Medical University of Białystok, 15-089 Białystok, Poland; sinemyiz.atalay@umb.edu.pl (S.A.); iwona.jarocka-karpowicz@umb.edu.pl (I.J.-K.)

**Keywords:** cannabidiol, cannabidiol synthetic derivatives, endocannabinoids, oxidative stress, lipid peroxidation, inflammation, membrane receptors

## Abstract

Cannabidiol (CBD) is one of the main pharmacologically active phytocannabinoids of *Cannabis sativa* L. CBD is non-psychoactive but exerts a number of beneficial pharmacological effects, including anti-inflammatory and antioxidant properties. The chemistry and pharmacology of CBD, as well as various molecular targets, including cannabinoid receptors and other components of the endocannabinoid system with which it interacts, have been extensively studied. In addition, preclinical and clinical studies have contributed to our understanding of the therapeutic potential of CBD for many diseases, including diseases associated with oxidative stress. Here, we review the main biological effects of CBD, and its synthetic derivatives, focusing on the cellular, antioxidant, and anti-inflammatory properties of CBD.

## 1. Introduction

The endocannabinoid system is an important molecular system responsible for controlling homeostasis and is becoming an increasingly popular target of pharmacotherapy. Endocannabinoids are ester, ether, and amide derivatives of long chain polyunsaturated fatty acids (PUFAs), such as arachidonic acid, and they act mainly as cannabinoid receptor ligands [1]. Endocannabinoids belong to a large group of compounds with a similar structure and biological activity called cannabinoids. Cannabinoids are chemical derivatives of dibenzopyrene or monoterpenoid, and to date over four hundred have been identified. The most important of these are Δ^9^-tetrahydrocannabinol (Δ^9^-THC), Δ^8^-tetrahydrocannabinol (Δ^8^-THC), cannabinol (CBN), and cannabidiol (CBD), and they are members of a large group of biologically active compounds found in *Cannabis sativa* L. [2]. The medical use of cannabinoids, in particular phytocannabinoids, has been one of the most interesting approaches to pharmacotherapy in recent years.

CBD is one of the main pharmacologically active phytocannabinoids [3]. It is non-psychoactive, but has many beneficial pharmacological effects, including anti-inflammatory and antioxidant effects. [4]. In addition, it belongs to a group of compounds with anxiolytic, antidepressant, antipsychotic, and anticonvulsant properties, among others [5]. The biological effects of cannabidiol, including the various molecular targets, such as cannabinoid receptors and other components of the endocannabinoid system, with which it interacts, have been extensively studied. The therapeutic potential of CBD has been evaluated in cardiovascular, neurodegenerative, cancer, and metabolic diseases, which are usually accompanied by oxidative stress and inflammation [6]. One of the best studied uses of CBD is for therapeutic effect in diabetes and its complications in animal and human studies [7]. CBD, by activating the cannabinoid receptor, CB2, has been shown to induce vasodilatation in type 2 diabetic rats [8,9], and by activating 5-HT_1A_ receptors, CBD showed a therapeutic effect in diabetic neuropathy [10]. Moreover, this phytocannabinoid accelerated wound healing in a diabetic rat model by protecting the endothelial growth factor (VEGF) [11]. In addition, by preventing the formation of oxidative stress in the retina neurons of diabetic animals, CBD counteracted tyrosine nitration, which can lead to glutamate accumulation and neuronal cell death [12].

This review summarizes the chemical and biological effects of CBD and its natural and synthetic derivatives. Particular attention was paid to the antioxidant and anti-inflammatory effects of CBD and its derivatives, bearing in mind the possibilities of using this phytocannabinoid to protect against oxidative stress and the consequences associated with oxidative modifications of proteins and lipids. Although CBD demonstrates safety and a good side effect profile in many clinical trials [4], all of the therapeutic options for CBD discussed in this review are limited in a concentration-dependent manner.

## 2. Molecular Structure of CBD

**Figure 1:**
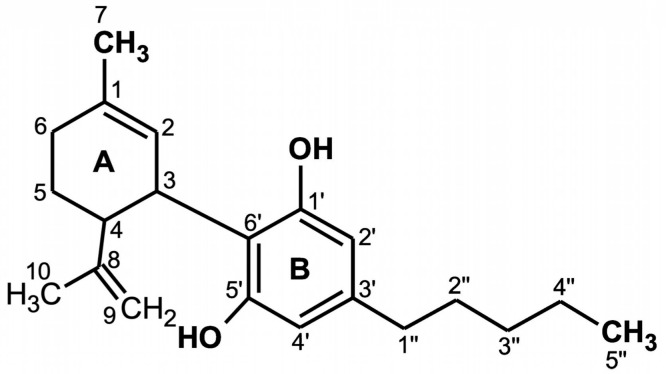
Chemical structure of cannabidiol (CBD) [16].

CBD is a terpenophenol compound containing twenty-one carbon atoms, with the formula C_21_H_30_O_2_ and a molecular weight of 314.464 g/mol (Figure 1). The chemical structure of cannabidiol, 2-[1R-3-methyl-6R-(1-methylethenyl)-2-cyclohexen-1-yl]-5-pentyl-1,3-benzenediol, was determined in 1963 [13]. The current IUPAC preferred terminology is 2-[(1R,6R)-3-methyl-6-prop-1-en-2-ylcyclohex-2-en-1-yl]-5-pentylbenzene-1,3-diol. Naturally occurring CBD has a (−)-CBD structure [14]. The CBD molecule contains a cyclohexene ring (A), a phenolic ring (B) and a pentyl side chain. In addition, the terpenic ring (A) and the aromatic ring (B) are located in planes that are almost perpendicular to each other [15]. There are four known CBD side chain homologs, which are methyl, *n*-propyl, *n*-butyl, and *n*-pentyl [16]. All known CBD forms (Table 1) have absolute *trans* configuration in positions 1R and 6R [16].

The CBD chemical activity is mainly due to the location and surroundings of the hydroxyl groups in the phenolic ring at the C-1′ and C-5′ positions (B), as well as the methyl group at the C-1 position of the cyclohexene ring (A) and the pentyl chain at the C-3′ of the phenolic ring (B). However, the open CBD ring in the C-4 position is inactive. Due to the hydroxyl groups (C-1′ and C-5′ in the B ring), CBD can also bind to amino acids such as threonine, tyrosine, glutamic acid, or glutamine by means of a hydrogen bond [17].

CBD has potential antioxidant properties because its free cationic radicals exhibit several resonance structures in which unpaired electrons are distributed mainly on ether and alkyl moieties, as well as on the benzene ring [18].

## 3. Biological Activity of CBD

CBD has a wide spectrum of biological activity, including antioxidant and anti-inflammatory activity, which is why its activity in the prevention and treatment of diseases whose development is associated with redox imbalance and inflammation has been tested [4,19,20]. Based on the current research results, the possibility of using CBD for the treatment of diabetes, diabetes-related cardiomyopathy, cardiovascular diseases (including stroke, arrhythmia, atherosclerosis, and hypertension), cancer, arthritis, anxiety, psychosis, epilepsy, neurodegenerative disease (i.e., Alzheimer’s) and skin disease is being considered [20,21,22]. Analysis of CBD antioxidant activity showed that it can regulate the state of redox directly by affecting the components of the redox system and indirectly by interacting with other molecular targets associated with redox system components.

### 3.1. Direct Antioxidant Effects of CBD

**Figure 2:**
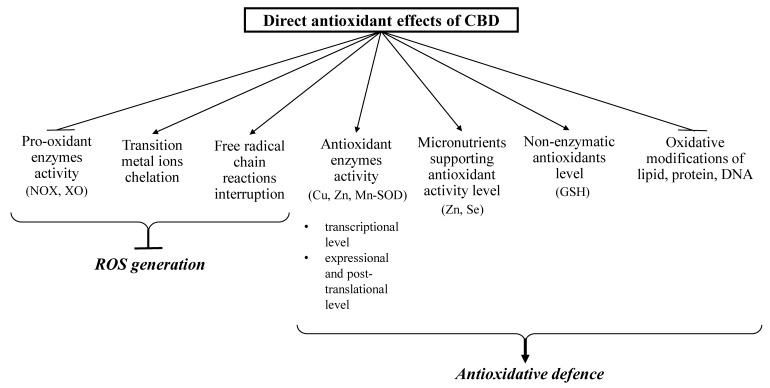
Direct antioxidant effects of CBD (closed arrows indicate reducing effects; opened arrows indicate inducing action).

**Figure 3:**
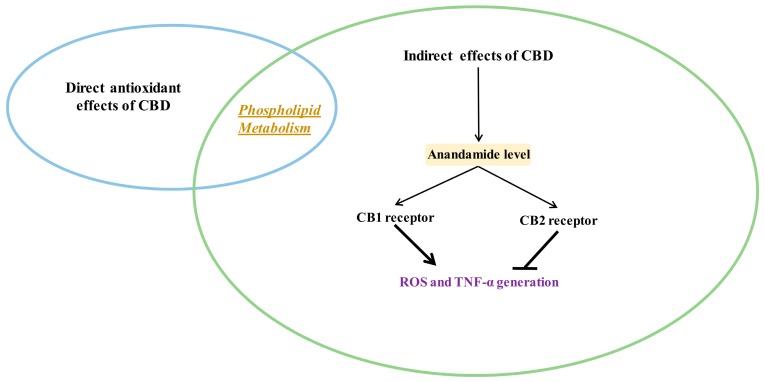
Indirect antioxidant and anti-inflammatory effects of CBD (closed arrows indicate inhibition; opened arrows indicate activation.

CBD has been shown to affect redox balance by modifying the level and activity of both oxidants and antioxidants (Figure 2 and Figure 3). CBD, like other antioxidants, interrupts free radical chain reactions, capturing free radicals or transforming them into less active forms. The free radicals produced in these reactions are characterized by many resonance structures in which unpaired electrons are mainly found on the phenolic structure, suggesting that the hydroxyl groups of the phenol ring are mainly responsible for CBD antioxidant activity [18].

CBD reduces oxidative conditions by preventing the formation of superoxide radicals, which are mainly generated by xanthine oxidase (XO) and NADPH oxidase (NOX1 and NOX4). This activity was shown in the renal nephropathy model using cisplatin-treated mice (C57BL/6J) [23] and in human coronary endothelial cells (HCAEC) [24]. In addition, CBD promoted a reduction in NO levels in the liver of doxorubicin-treated mice [25] and in the paw tissue of Wistar rats in a chronic inflammation model [26].

CBD also reduces reactive oxygen species (ROS) production by chelating transition metal ions involved in the Fenton reaction to form extremely reactive hydroxyl radicals [27]. It was shown that CBD, acting similarly to the classic antioxidant butylated hydroxytoluene (BHT), prevents dihydrorodamine oxidation in the Fenton reaction [28]. In addition, CBD has been found to decrease β-amyloid formation in neurons by reducing the concentration of transition metal ions [29]. 

In addition to the direct reduction of oxidant levels, CBD also modifies the redox balance by changing the level and activity of antioxidants [19,26]. CBD antioxidant activity begins at the level of protein transcription by activating the redox-sensitive transcription factor referred to as the nuclear erythroid 2-related factor (Nrf2) [30], which is responsible for the transcription of cytoprotective genes, including antioxidant genes [31]. CBD was found to increase the mRNA level of superoxide dismutase (SOD) and the enzymatic activity of Cu, Zn- and Mn-SOD, which are responsible for the metabolism of superoxide radicals in the mouse model of diabetic cardiomyopathy type I and in human cardiomyocytes treated with 3-nitropropionic acid or streptozotocin [32]. Repeated doses of CBD in inflammatory conditions were found to increase the activity of glutathione peroxidase and reductase, resulting in a decrease in malonaldehyde (MDA) levels, which were six times higher in untreated controls [26]. Glutathione peroxidase activity (GSHPx) and glutathione level (GSH) were similarly changed after using CBD to treat UVB irradiated human keratinocytes. The high affinity of CBD for the cysteine and selenocysteine residues of these proteins is a possible explanation for this observation [33]. It is known that under oxidative conditions, alterations in enzymatic activity may be caused by oxidative modifications of proteins, mainly aromatic and sulfur amino acids [34]. It has also been suggested that the reactive CBD metabolite cannabidiol hydroxyquinone reacts covalently with cysteine, forming adducts with, for example, glutathione and cytochrome P450 3A11, and thereby inhibiting their biological activity [35]. In addition, CBD has been found to inhibit tryptophan degradation by reducing indoleamine-2,3-dioxygenase activity [36]. CBD also supports the action of antioxidant enzymes by preventing a reduction in the levels of microelements (e.g., Zn or Sn), which are usually lowered in pathological conditions. These elements are necessary for the biological activity of some proteins, especially enzymes such as superoxide dismutase or glutathione peroxidase [25]. 

By lowering ROS levels, CBD also protects non-enzymatic antioxidants, preventing their oxidation, as in the case of GSH in the myocardial tissue of C57BL/6J mice with diabetic cardiomyopathy [32] and doxorubicin-treated rats [25]. An increase in GSH levels after CBD treatment was also observed in mouse microglia cells [37] and in the liver of cadmium poisoned mice [25]. This is of great practical importance because GSH cooperates with other low molecular weight compounds in antioxidant action, mainly with vitamins such as A, E, and C [38]. CBD exhibits much more antioxidant activity (30–50%) than α-tocopherol or vitamin C [4].

### 3.2. The Consequences of Direct Antioxidant Action of CBD

The result of an imbalance between oxidants and antioxidants is oxidative stress, the consequences of which are oxidative modifications of lipids, nucleic acids, and proteins. This results in changes in the structure of the above molecules and, as a result, disrupts their molecular interactions and signal transduction pathways [39]. Oxidative modifications play an important role in the functioning of redox-sensitive transcription factors (including Nrf2 and the nuclear factor kappa B (NFκB). As a consequence, oxidative modifications play a role in the regulation of pathological conditions characterized by redox imbalances and inflammation, such as cancer, inflammatory diseases, and neurodegenerative diseases [40,41]. 

In this situation, one of the most important processes is lipid peroxidation, which results in the oxidation of polyunsaturated fatty acids (PUFA), such as arachidonic, linoleic, linolenic, eicosapentaenoic, and docosahexaenoic acids [42]. As a result of the ROS reaction with PUFAs, lipid hydroperoxides are formed, and as a result of oxidative fragmentation, unsaturated aldehydes are generated, including 4-hydroxynenenal (4-HNE), malonodialdehyde (MDA) or acrolein [43]. In addition, the propagation of oxidation chain reactions, especially with regard to docosahexaenoic acid, can lead to oxidative cyclization, resulting in production of isoprostanes or neuroprostanes [44]. The formation of lipid peroxidation products directly affects the physical properties and functioning of the cell membranes in which they are formed [42]. Due to their structure (the presence of a carbonyl groups and carbon-carbon double bonds) and electrophilic character, generated unsaturated aldehydes are chemically reactive molecules that can easily form adducts with the majority of the cell’s nucleophilic components, including DNA, lipids, proteins, and GSH [45]. For example, 4-hydroxynonenal (4-HNE) has been identified as a stimulator of the cytoprotective transcription factor Nrf2, an inhibitor of antioxidant enzymes (e.g., catalase and thioredoxin reductase) and a pro-inflammatory factor acting through the NFκB pathway [46]. These reactions reduce the level of reactive lipid peroxidation products, while increasing the formation of adducts with proteins that promote cell signaling disorders, thus stimulating metabolic modifications that can lead to cellular dysfunction and apoptosis [47,48]. 

In addition to lipid peroxidation, oxidative conditions also favor the oxidative modification of proteins by ROS. The aromatic and sulfhydryl amino acid residues are particularly susceptible to modifications, and can result in production of levodopa (l-DOPA) from tyrosine, ortho-tyrosine from phenylalanine, sulfoxides and disulfides from cysteine, and kynurenine from tryptophan, among others [49]. The resulting changes in the protein structures cause disruption of their biological properties and, as in the case of lipid modification, affect cell metabolism, including signal transduction [46,50].

One of the most noticeable CBD antioxidant effects is the reduction in lipid and protein modifications [25,51]. CBD supplementation has been found to reduce lipid peroxidation, as measured by MDA levels, in mouse hippocampal (HT22) neuronal cells depleted of oxygen and glucose under reperfusion conditions [51]. A reduction in lipid peroxidation following CBD supplementation has also been shown in C57BL/6J mouse liver homogenates, assessed by 4-HNE levels [52]. CBD also protected the brain against oxidative protein damage caused by D-amphetamine in a rat model of mania [53]. On the other hand, CBD induced ubiquitination of the amyloid precursor protein (APP), an indicator of cellular changes in the brain of people with Alzheimer’s disease, when evaluated in human neuroblastoma cells (SHSY5Y^APP+^) [54]. In addition, CBD treatment has recently been shown to exhibit an unusual protective effect by transporting proteins including multidrug-1 resistance protein and cytosol transferases, such as S-glutathione-M1 transferase, prior to modification by lipid peroxidation products. This prevents elevation of 4-HNE and MDA adduct levels in fibroblast cell culture [55]. It was also shown that this phytocannabinoid reduced the level of small molecular αβ-unsaturated aldehydes in the myocardial tissue of Sprague-Dawley rats and mice with diabetic cardiomyopathy, and in the liver of mice from the acute alcohol intoxication model [21,25,32]. Additionally, CBD caused a reduction in the level of PUFA cyclization products, such as isoprostanes, in the cortex of transgenic mice (APPswe/PS1ΔE9) with Alzheimer’s disease [56]. Thus, CBD protects lipids and proteins against oxidative damage by modulating the level of oxidative stress, which participates in cell signaling pathways. 

### 3.3. Indirect Antioxidant Effects of CBD

**Figure 4:**
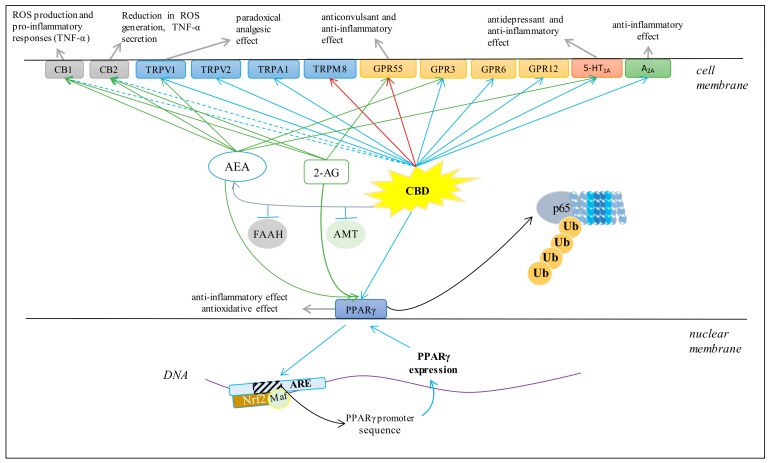
Major effects of CBD on several membrane receptors (AEA, anandamide; 2-AG, 2-arachidonoylglycerol; FAAH, fatty acid amide hydrolase; AMT, AEA membrane transporter; ROS, reactive oxygen species; Ub, ubiquitin; p65, transcription factor NF-κB; Nrf2, nuclear factor erythroid 2-related factor 2; ARE, antioxidant response elements. Blue arrows indicate agonist activity; red arrows indicate antagonist activity; dashed blue arrows indicate weakly agonistic activity; green arrows indicate endocannabinoid agonist activity; grey arrows indicate chemical and biological effects).

Various cell metabolic systems, including the endocannabinoid system, are involved in the regulation of redox balance. Thus, the action of CBD as a phytocannabinoid may support the biological activity of the endocannabinoid system. CBD has recently been shown to modulate the endocannabinoid system activity by increasing anandamide (AEA) levels [5], which can affect cannabinoids signaling, including their interaction on cannabinoid receptors [57]. However, it is known that the peroxisome proliferator-activated receptor alpha (PPAR-α), for example, activated by endocannabinoids, directly regulates the expression of antioxidant enzymes such as superoxide dismutase by interacting with their promoter regions [58]. Therefore, it is believed that the most important antioxidant activity of CBD, like endocannabinoids, is associated with its effect on receptors. CBD, depending on the concentration, can activate, antagonize or inhibit cannabinoid receptors (CB1 and CB2), as well as ionotropic (TRP) and nuclear (PPAR) receptors (Figure 4) [52,59,60]. 

### 3.4. Cannabinoid Receptors

CBD has been shown to be a weak agonist of the human, mouse, and rat CB1 receptor [61]. The activation of the CB1 receptor increases ROS production and a pro-inflammatory response, including the downstream synthesis of tumor necrosis factor α (TNF-α) [62]. In addition, it was shown that CBD is a negative allosteric modulator of the CB1 receptor [63]. Regardless of the effect on the CB1 receptor, CBD is a weak agonist of the CB2 receptor [64], but it has also been suggested that it may demonstrate inverse agonism of the CB2 receptor [65]. Importantly, CB2 activation leads to a decrease in ROS and TNF-α levels, which reduces oxidative stress and inflammation [62]. Therefore, it has been suggested that CBD may indirectly improve anti-inflammatory effects. Clinical studies have confirmed that CBD reduces the levels of pro-inflammatory cytokines, inhibits T cell proliferation, induces T cell apoptosis and reduces migration and adhesion of immune cells [66]. In addition, CBD anti-inflammatory activity has been shown to be antagonized by both a selective CB2 antagonist and AEA, an endogenous CB2 receptor agonist [67]. 

CB1 and CB2 are receptors with strong expression in the central nervous system and the immune system primarily, but also occur in other tissues. CBD, acting on the above receptors, inhibits the activity of adenylyl cyclase and voltage gated calcium channels, activates potassium channels and activates mitogen activated protein kinase (MAPK), 3-phosphoinositol kinase (PI3K)/AKT, and the mammalian target of rapamycin (mTOR) signaling pathways [68]. The PI3K/AKT/mTOR pathway is one of the basic pathways necessary for physiological protein synthesis and induction of other intracellular pathways, such as the MAPK pathway, which plays an important role in regulating cell survival, proliferation, and apoptosis [69]. CBD was found to induce apoptosis in leukemia cells by reducing p38-MAPK levels [70]. However, CBD was also shown to inhibit apoptosis in human breast cancer cell lines (T-47D and MDA-MB-231) by inhibiting expression of oncogenic and pro-survival cyclin D1 and mTOR, and by increasing PPARγ receptor expression [71]. 

### 3.5. TRP Receptors 

It has been shown that CBD can also affect redox balance and inflammation by modulating mammalian transient receptor potential (TRP) channels [72,73]. CBD activates vanilloid receptors (TRPV), directly or indirectly, by increasing the level of endogenous AEA, which is one of the endogenous TRPV1 agonists [64]. CBD, as a TRPV1 receptor agonist, binds to it and causes desensitization, leading to “paradoxical analgesic activity” similar to that of capsaicin [26]. It has been suggested that there is a relationship between molecular signaling of TRPV1 and oxidative stress [74] because ROS and lipid peroxidation products can regulate the physiological activity of TRPV1 by oxidizing its thiol groups [75]. Consequently, CBD not only activates TRP through a direct agonist-receptor interaction, but also by lowering the level of oxidative stress. In addition, CBD activates other vanilloid receptors such as TRPV2 and the potential ankarin protein 1 receptor subtype (TRPA1), while antagonizing the TRP-8 receptor (TRPM8) [72]. CBD has also been shown to stimulate calcium ions in transfected HEK-293 cells via TRPV3 [76] and regulate calcium ion homeostasis in immune and inflammatory cells mainly via TRP channels, which is important for proliferation and pro-inflammatory related cytokine secretion [77]. In addition, Ca^2+^ ions control the activation of several transcription factors (e.g., NFAT) that regulate the expression of various cytokines, such as IL-2, IL-4 and IFNγ, which affect cellular inflammatory responses [78].

Regardless of the direct effect of CBD on TRP receptors, increasing the level of AEA, as a full TRPV1 agonist, also affects the activation of TRP receptors and negatively regulates the 2-arachidonoylglycerol (2-AG) metabolism [79]. It has been shown that both AEA and 2-AG can be synthesized in the plasma membrane. However, the degradation of phosphatidylinositol by phospholipase C results in the formation of a diacylglycerol precursor, whose hydrolysis (through diacylglycerol lipase activity, DAGL) allows the formation of 2-AG [80]. However, activation of DAGLα and DAGLβ requires GSH. Additionally, these enzymes are sensitive to Ca^2+^ ions [81]. TRPV1 agonists, such as capsaicin and AEA, have been shown to inhibit 2-AG synthesis in striatal neurons of C57BL/6 mice by glutathione-dependent pathways, since DAGL is stimulated by GSH [82]. In addition, the interaction between AEA and 2-AG has been shown to disappear after inactivation of TRPV1 channels. This suggests that the negative effect of AEA on 2-AG metabolism can be mimicked by stimulation of TRPV1 channels. Therefore, AEA and 2-AG interactions require redox balance, due to the participation of GSH in 2-AG synthesis. In summary, CBD modifies TRPV1 receptor activation through reducing oxidative stress as well as biosynthesis of 2-AG.

### 3.6. PPARγ Receptor

CBD is an agonist of the PPARγ receptor, which is a member of the nuclear receptor superfamily of ligand-inducible transcription factors [52]. PPARγ, an ubiquitin E3 ligase, has been shown to interact directly with NFκB. The interaction occurs between the ligand-binding domain of PPARγ and the Rel homology domain region of the p65 subunit of NFκB. Lys48-linked polyubiquitin of the ligand-binding domain of PPARγ is responsible for proteosomal degradation of p65 [83]. In this way, PPARγ participates in the modulation of inflammation by inducing ubiquitination proteosomal degradation of p65, which causes inhibition of pro-inflammatory gene expression, such as cyclooxygenase (COX2) and some pro-inflammatory mediators such as TNF-α, IL-1β, and IL-6, as well as inhibition of NFκB-mediated inflammatory signaling [84]. For this reason, PPARγ agonists can play an anti-inflammatory role by inhibiting the NFκB-mediated transcription of downstream genes [84]. This molecular mechanism is mediated by β-catenin and glycogen synthase kinase 3 beta (GSK-3β). β-catenin attenuates transcription of pro-inflammatory genes by inhibiting NFκB [85,86]. On the other hand, GSK-3β is decreased by PPARγ stimulation [87]. 

PPARγ cooperates also with another transcription factor, Nrf2, which controls the expression of genes encoding cytoprotective proteins, particularly antioxidant proteins [28,88]. PPARγ may bind to specific elements in the promoter region of genes it regulates, including Nrf2, catalase (CAT), glutathione *S*-transferase (GST), heme-oxygenase-1 (HO-1), and manganese-dependent superoxide dismutase (Mn-SOD). In contrast, Nrf2 can regulate PPARγ expression by binding to the PPARγ promoter in the sequence of antioxidant response elements (ARE) that are located in the -784/-764 and -916 regions of the PPARγ promoter [89,90]. The reduction in PPARγ expression in Nrf2 knockout mice provides confirmation of this regulation [91].

Acting through the PPARγ receptor, CBD demonstrates anti-inflammatory and antioxidant properties. In addition, direct CBD activity is enhanced by the action of AEA and 2-AG, which are also PPARγ agonists and whose levels are elevated by CBD [92]. It has been found that stimulation of PPARα and reduction of oxidative stress by CBD prevents amyloid β-induced neuronal death by increasing the levels of Wnt/β-catenin [84]. However, there are no data on the interaction between CBD and other PPAR subtypes (PPARα, β, δ). It is known that the endocannabinoids AEA (whose biosynthesis is stimulated by CBD) and 2-AG can activate PPARγ [92]. AEA activates PPARα, while the 2-AG derivative 15-hydroxyyeicosatetraenoic acid glyceryl ester increases the transcriptional activity of PPARα [92]. In summary, CBD demonstrates anti-inflammatory activity and antioxidant effects by activating PPARs, either directly or indirectly.

### 3.7. GPR Receptors

GPR55, which is strongly expressed in the nervous and immune systems as well as in other tissues, is a G-protein coupled receptor [93]. Activation of GPR55 increases the intracellular level of calcium ions [94]. CBD is a GPR55 antagonist and can modulate neuronal Ca^2+^ levels depending on the excitability of cells [95]. CBD antagonism is manifested as an anticonvulsant effect [96]. Because CBD increases endocannabinoid expression, it can also indirectly affect inflammation and redox balance via these molecules [58]. In addition, GPR55 knockout mice have been shown to have high levels of anti-inflammatory interleukins (IL-4, IL-10, and IFN-γ) [97], while high expression of GPR55 reduces ROS production [98]. Therefore, the organism’s response to CBD depends on whether direct or indirect effects dominate.

CBD has also been shown to be an inverse agonist of other GPR receptors, including GPR3, GPR6 and GPR12. It reduces β-arestinin 2 levels and cAMP accumulation in amyloid plaque formation in the development of Alzheimer’s disease, in a concentration-dependent manner [98]. In addition, one of the neuropharmacological effects of CBD is its reducing effect on hippocampal synaptosomes mediated by its interaction with GPR3 [99]. It has also been suggested that the effect of CBD on these orphan receptors represents a new therapeutic approach in diseases such as Alzheimer’s disease, Parkinson’s disease, cancer, and infertility [100].

### 3.8. 5-HT_1A_ Receptor

CBD has direct affinity for the human 5-HT_1A_ (serotonin) receptor [101]. In addition, CBD can induce the 5-HT_1A_ receptor indirectly by increasing the level of AEA [102]. However, the activated 5-HT_1A_ receptor can act as a membrane antioxidant by capturing ROS [103]. Therefore, through activation of 5-HT_1A_, CBD can counteract peroxidation of phospholipids and thus participate in the protection of biomembranes against oxidative modifications. In addition, studies in Wistar rats have shown that CBD, by activating 5-HT_1A_ receptors, can reduce physiological and behavioral responses to restrictive stress [104]. CBD has also been suggested as a therapeutic compound for the treatment of painful diabetic neuropathy due to its ability to activate 5-HT_1A_ receptors [10].

### 3.9. Adenosine A_2A_ Receptors

CBD is also an agonist of adenosine A_2A_ receptors [61], which are G-protein coupled receptors. They are expressed in various cell types, participate in numerous physiological and pathological processes and also regulate inflammatory processes [105]. Adenosine and its agonists exhibit anti-inflammatory activity in vivo [106]. Therefore, adenosine release is one of the mechanisms of immunosuppression during inflammation [107], and adenosine receptor agonists reduce TNF-α levels [108,109]. It has been shown that CBD by activating A_2A_ adenosine receptors can reduce the level of vascular cell adhesion molecule (VCAM-1) in endothelial cells in SJL/J mice, which may provide a new mechanism to control neuroinflammatory diseases such as multiple sclerosis (MS) [110]. 

In addition, it has been found that A_2A_ activation can prevent reperfusion consequences and alleviate oxidative stress in mitochondria [111]. This suggests that CBD prevents oxidative stress by activating A_2A_ receptors. It was also shown that A_2A_ receptors can form heteromers with CB1 receptors in CA1 neurons and in the hippocampus of C57BL/6J mice [112]. Therefore, CBD can modify the functioning of the entire heteromer, and thus modulate the activation of two groups of receptors involved in the regulation of redox balance and inflammation.

## 4. Effects of Natural Derivatives of CBD on Receptors

Due to the range of CBD metabolic effects known to date, interest in the possibility of using this phytocannabinoid is constantly growing. Considering the fact that modifications to the CBD structure may result in an improved therapeutic profile and biological activity, natural CBD derivatives are sought and their therapeutic utility is being evaluated. Therefore, known or potential effects of naturally occurring CBD derivatives are presented. Their activity through membrane receptors is emphasized, which are described in this review as those which under the influence of CBD show antioxidant and/or anti-inflammatory activities.

### 4.1. CB1/CB2 Receptors

Cannabidiolic acid (CBDA), being a C3′-carboxyl derivative of CBD (2,4-dihydroxy-3-[(1R, 6R)-3-methyl-6-prop-1-en-2-ylcyclohex-2-en-1-[alpha]-6-pentylbenzoic acid), acts as a selective COX2 and prostaglandin endoperoxide synthase inhibitor and exhibits anti-inflammatory properties in human breast cancer cells [113]. It has been suggested that its action may be due to a weak affinity for CB1 and CB2 receptors (Table 2) [114]. Similarly, other CBD derivatives, such as cannabidivarin (CBDV), which is a CBD analogue of C4′-propyl (2-[(1R, 6R)-3-methyl-6-prop-1-en-2)-ylcyclohex-2-en-1-yl]-5-propylbenzene-1,3-diol), 7-hydroxy-CBD (7-OH-CBD) and the hydroxylated CBD derivative of 7-carboxylic acid (7-COOH-CBD) have poor affinities for CB1 and CB2 (Table 2) [2]. There is no published data examining their impact on the redox balance.

### 4.2. GPR55 and TRPV1 Receptors

CBDV has been found to have antagonistic effects on GPR55 (Table 2), which probably leads to anticonvulsant effects [115]. Therefore, this compound is suggested for use when therapeutic antiepileptic activities are needed. On the other hand, CBDA has been suggested to be an effective compound in analgesia and cancer through its agonistic action on TRPA1 and TRPV1 receptors (Table 2) and antagonistic action on TRPM8, similar to CBD [76,116].

Another natural phytocannabinoid is cannabimovone (1-[(1R, 2R, 3R, 4R)-3-(2,6-dihydroxy-4-pentylphenyl)-2-hydroxy-4-prop-1-en-2-ylcyclopentyl] ethanone), which has low affinity for the CB1 and CB2 receptors, but significant affinity for TRPV1 (Table 2) [117]. 

In contrast, cannabigivarin (a cannabigerol ropyl analogue) has been shown to stimulate and desensitize human TRPV1 (Table 2) [72]. It is also known that TRPV1 receptor activity is deeply involved in oxidative stress and inflammation [114]. Based on the understanding of the relationship between TRPV1 and oxidative stress described in Section 3.5, all of these derivatives may provide different therapeutic approaches in the case of inflammation and oxidative stress.

### 4.3. 5-HT_1A_ and PPARγ Receptors

It has been shown that another CBD derivative, cannabigerol (CBG; (2-[(2E)-3,7-dimethylocta-2,6-dienyl]-5-pentylbenzene-1,3-diol]), a naturally open analogue of cyclohexenyl CBD, activates TRPV1 as well as 5-HT_1A_ (Table 2) and has antidepressant and anti-inflammatory effects in intestinal diseases [2,72,118]. CBG may also bind to PPARγ (Table 2) and increase its transcriptional activity [92]. Studies on the HEK293 cell line have shown that CBG, by activating PPARγ, significantly reduces the secretion of inflammatory mediators such as IL-6 and TNF-α [119].

## 5. Effects of Synthetic Derivatives of CBD on Receptors

Given the limitations in the biological activity of CBD itself and its natural derivatives and the fact that the biological properties of CBD derivatives depend on their structure, synthetic derivatives are produced that have been designed so that their structure allows direct interaction with components of the redox system or indirectly with molecular targets interacting with these components, including the cannabinoid receptors (Table 2). The derivatives with potential antioxidant and anti-inflammatory effects include, but are not limited to, (+)-CBD derivatives, dihydrocannabidiol and tetrahydrocannabidiol derivatives, and (+)-dihydro-7-hydroxy-CBD [2]. Promising synthetic derivatives that can modulate redox balance and/or inflammation are presented below.

### 5.1. CB1/CB2 Receptors 

It has been shown that both the naturally occurring (−)-CBD enantiomer and its synthetic derivatives [(−)-7-hydroxy-5′-dimethylheptyl-CBD, and (−)-1-COOH-5′-dimethylheptyl-CBD] have weak affinity for the CB1 and CB2 cannabinoid receptors (Table 2). However, (+)-CBD and its derivatives [(+)-5′-dimethylheptyl-CBD and (+)-7-hydroxy-5′-dimethylheptyl-CBD] have high CB1 receptor affinity, slightly lower affinity for the CB2 receptor and inhibit AEA cellular uptake [120]. Similarly, (−)-7-hydroxy-dimethylheptyl-CBD can inhibit both AEA uptake and degradation through fatty acid amide hydrolase (FAAH) activity [121]. Recently, (−)-dimethylheptyl-CBD has been shown to be a CB1 receptor agonist (Table 2) in HEK-293A cells [122]. In addition, by reducing the expression of pro-inflammatory genes (IL-1b, IL-6, and TNF-α), it exhibits a dose-dependent anti-inflammatory effect on microglia BV-2 cells [30].

Furthermore, hydrogenated CBD derivatives such as (+)-dihydrocannabidiol and (+)-tetrahydrocannabidiol have CB1 receptor affinity (Table 2) and show anti-inflammatory effects on the peritoneal cells of C57BL/6 mice and a macrophage cell line. This behavior may suggest that the activation of pro-inflammatory mediators is not directly through the CB1 cannabinoid receptor [123]. Similarly, the (+)-8,9-dihydro-7-hydroxy-CBD derivative (HU-465), which has anti-inflammatory activity, especially at higher concentrations, binds to both CB1 and CB2 receptors, while its (−) enantiomer, (−)-8,9-dihydro-7-hydroxy-CBD (HU-446) has negligible affinity for both CB1 and CB2 receptors (Table 2). However, both HU-465 and HU-446 have been found to exhibit anti-inflammatory activity by inhibiting the release of IL-17 in mouse encephalitogenic T cells (TMOG) [124].

In addition, the pinene dimethoxy-dimethylheptyl-CBD derivative HU-308 [(3R, 4S, 6S)-2-[2,6-dimethoxy-4-(2-methyloctan-2-yl)phenyl]-7,7-dimethyl-4-bicyclo[3.1.1]hept-3-enyl]methanol] and its enantiomer HU-433 [(3S, 4R, 6R)-2-[2,6-dimethoxy-4-(2-methyloctan-2-yl)phenyl]-7,7-dimethyl-4-bicyclo[3.1.1]hept-3-enyl]methanol] were shown to have specific agonistic activity for the CB2 receptor (Table 2), and consequently, anti-inflammatory activity in cultured calvarial osteoblasts from C57BL/6J mice [125]. However, it has been found that HU-433 exhibits greater anti-inflammatory activity with poorer CB2 receptor binding affinity (Table 2) [125]. In contrast, HU-308, a CB2 agonist, was found to decrease TNF-α-induced expression of ICAM-1 and VCAM-1 in sinusoidal endothelial cells of human liver tissue [24]. Another CB2 receptor agonist, HU-910 ((1S,4R)-2-[2,6-dimethoxy-4-(2-methyloctan-2-yl)phenyl]-7,7-dimethyl-1-bicyclo[2.2.1]hept-2enyl]methanol)), significantly inhibits the effects of LPS that lead to increased inflammation (assessed by increased TNF-α expression) and increased oxidative stress (assessed by increased levels of 4-HNE and protein carbonyl groups) in mouse Kupffer cells [126]. This suggests that these effects are associated with CB2 receptor activation (Table 2).

### 5.2. GPR Receptors

Abnormal CBD (4-[(1R,6R)-3-methyl-6-prop-1-en-2-ylcyclohex-2-en-1-yl]-5-pentylbenzene-1,3-diol), named O-1602, is a synthetic CBD regioisomer and a selective GPR55 receptor agonist (Table 2), but not a CB1/CB2 receptor agonist. It causes vasodilation independent of the receptors as well [2,127]. Therefore, it has been suggested that O-1602 can be used to regulate ROS/RNS levels and modify the effect of oxidative stress on cellular metabolism by modulating GPR55 receptor activation [58]. In addition, O-1602, as a GPR18 agonist, mediates the reduction of cyclic adenosine monophosphate (cAMP) and the activation of the PI3K/AKT and ERK1/2 pathways in vitro [128]. A Sprague-Dawley rat study showed that GPR18 receptor activation via O-1602 leads to reduction of ROS levels, however inhibition of GPR18 receptor activation increases oxidative stress by increasing ROS production [129]. 

### 5.3. PPAR γ Receptor 

Recently it has also been found that the synthetic quinoline derivatives of CBG, VCE-003 [(2-[(2E)-3,7-dimethylocta-2,6-dienyl]-3-hydroxy-5-pentylcyclohexa-2,5-dien-1,4-dione)] and HU-331 [(3-hydroxy-2-[(1R,6R)-3-methyl-6-prop-1-en-2-ylcyclohex-2-en-1-yl]-5-pentylcyclohexa-2,5-dien-1,4-dione], activate the PPARγ receptor as CBG does (Table 2) [130]. HU-311 was shown to interfere with mitochondrial transmembrane potential and induce ROS generation, as well as activate the Nrf2 pathway [130]. However, another CBD derivative, VCE-003, induces PPARγ-mediated antioxidant and anti-inflammatory activity that prevents neuronal damage caused by inflammation in the Parkinson’s mouse model (intravascular LPS injection). The same effect was seen in the in vitro cellular model of neurological inflammation (BV2 cells exposed to LPS and M-213 cells treated with media prepared from BV2 cells exposed to LPS) [131].

### 5.4. TRPV1, 5-HT_1A_ and Adenosine A_2A_ Receptors

Despite extensive research into the biological effects of synthetic CBD derivatives, they have not been evaluated for their interaction with TRPV1, 5-HT_1A_ and adenosine A_2A_ receptors in the context of anti-inflammatory and antioxidant activity.

## 6. Conclusions

Oxidative stress resulting from overproduction of ROS is a key element of the immune system’s response to combat pathogens and initiates tissue repair. However, metabolic modifications resulting from overproduction of ROS also have many negative aspects and lead to the development and/or exacerbation of many diseases. It is believed that the endocannabinoid system, which includes G-protein coupled receptors and their endogenous lipid ligands, may be responsible for the therapeutic modulation of oxidative stress in various diseases. In this context, the phytocannabinoid cannabidiol, which was identified several decades ago and may interact with the cannabinoid system, is a promising molecule for pharmacotherapy.

Relatively recently, multidirectional biological effects have been demonstrated in various preclinical models, including the antioxidant and anti-inflammatory effects of cannabidiol [14,73]. In the context of the above data, CBD seems to be more preferred than other compounds from the phytocannabinoid group. Regardless of the beneficial pharmacological effects of CBD itself, if this compound is present in the Δ^9^-THC environment, the undesirable effects of 99-THC are reduced, which improves its safety profile [132].

Important in CBD therapeutic applications is the lack of psychotropic effects. Furthermore, this phytocannabinoid is not teratogenic or mutagenic [133]. Until recently, CBD was thought to have only low toxicity to humans and other species [134], but recent studies indicate an increase in ALT and AST levels after CBD treatment, which disqualifies it as the drug of choice [135,136]. In addition, it has been found that CBD may interfere with the hepatic metabolism of some drugs by inactivating cytochrome P450 3A and P450 2C [137]. Such interactions should be considered when co-administering CBD with other drugs metabolized by above enzymes.

In order to find compounds with a greater therapeutic profile and activity than CBD, without any adverse effects, the biological properties of both natural and synthetic CBD derivatives were checked, with the hope of finding the perfect derivative that provides a close to ideal therapeutic effect.

## Figures and Tables

**Table 1:** Cannabidiol derivatives [16].

Compound	R^1^	R^2^	R^3^
cannabidiolic acid (CBDA-C_5_)	COOH	n-C_5_H_11_	H
(–)-cannabidiol (CBD-C_5_)	H	n-C_5_H_11_	H
cannabidiol monomethyl ether (CBDM-C_5_)	H	n-C_5_H_11_	Me
Cannabidiol-C_4_ (CBD-C_4_)	H	n-C_4_H_9_	H
cannabidivarinic acid (CBDVA-C_3_)	COOH	n-C_3_H_7_	H
(–)-cannabidivarin (CBDV-C_3_)	H	n-C_3_H_7_	H
cannabidiorcol (CBD-C_1_)	H	CH_3_	H

**Table 2:** Influence of natural and synthetic CBD derivatives on receptor activation (X: agonist activation or Y: antagonist activation by related CBD derivative; * weak affinity; #: full name is in chapter 4.1) [2,24,72,76,114,115,116,120,122,123,124,125,126,127,130].

CBD Derivatives	Membrane Receptors								
Natural	CB1	CB2	Gpr55	Gpr18	TRPV1	TRPA1	TRPM8	5-HT_1A_	PPARγ
cannabigerol (CBG)					X			X	
cannabigivarin (CBGV)					X				
cannabidiolic acid (CBDA)	X *	X *			X	X	Y		
cannabidivarin (CBDV)	X *	X *	Y						
cannabimovone	X *	X *			X				
7-OH-CBD	X *	X *							
7-COOH-CBD	X *	X *							
**Synthetic**	**CB1**	**CB2**	**Gpr55**	**Gpr18**	**TRPV1**	**TRPA1**	**TRPM8**	**5-HT_1A_**	**PPARγ**
(−)-dimethylheptyl-CBD (DMH-CBD)	X								
(−)-7-hydroxy-5′-dimethylheptyl-CBD	X *	X *							
(−)-1-COOH-5′-dimethylheptyl-CBD	X *	X *							
(+)-5′-dimethylheptyl-CBD	X	X *							
(+)-7-hydroxy-5′-dimethylheptyl-CBD	X	X *							
(+)-dihydrocannabidiol (H2-CBD)	X								
(+)-tetrahydrocannabidiol (H4-CBD)	X								
(-)-8,9-dihydro-7-hydroxy-CBD (HU-446)	X *	X *							
(+)-8,9-dihydro-7-hydroxy-CBD (HU-465)	X	X							
pinene dimethoxy-dimethylheptyl-CBD derivative (HU-433)		X *							
pinene dimethoxy-dimethylheptyl-CBD derivative (HU-308)		X							
HU-910 #		X							
4′-fluorocannabidiol (HUF-101/4′-F-CBD)	X	X							
quinol derivative VCE-003									X
quinol derivative HU-331									X
abnormal-CBD			X	X					
